# Barriers and Factors Affecting Nursing Communication When Providing Patient Care in Jeddah

**DOI:** 10.3390/clinpract15010019

**Published:** 2025-01-14

**Authors:** Ruba M. Alharazi, Rahaf J. Abdulrahim, Alhanouf H. Mazuzah, Reem M. Almutairi, Hayfa Almutary, Aisha Alhofaian

**Affiliations:** 1 Medical Surgical Nursing Department, Faculty of Nursing, King Abdulaziz University, Jeddah 21589, Saudi Arabiaaalhofaian@kau.edu.sa (A.A.); 2 Faculty of Nursing, King Abdulaziz University, Jeddah 21589, Saudi Arabiaamazuzah@stu.kau.edu.sa (A.H.M.);

**Keywords:** communication, barrier, nurses, culture, care, patients

## Abstract

**Background/Objectives:** Effective communication between nurses and patients plays a crucial role in the delivery of quality healthcare services, especially when caring for patients from diverse cultural backgrounds. It fosters trust, understanding, and collaboration and contributes to better health outcomes and satisfactory nurse–patient relationships. The aim of the study is to assess the factors and barriers affecting nurses’ communication when providing care for patients from diverse cultural backgrounds in Jeddah, Saudi Arabia. **Methods:** A cross-sectional quantitative descriptive design is used with an online survey instrument. The study involved registered nurses employed in Jeddah’s hospitals. The study utilized a convenience sample for data collection and used the latest version of the statistical package for the social sciences (SPSS version 21) for data entry and analysis. **Results:** A study of 367 participants found significant barriers to nurse–patient communication, with a mean score of 2.84 on a three-point scale. Key challenges included language differences between nurses and patients with a mean score of 2.87, and cultural and religious differences with a mean score of 2.83 and 2.81, as well as nurses’ communication skills, attitudes, and self-confidence and patients’ awareness, attitudes, and resistance to communication. The multifaceted nature of these barriers emphasizes the need for targeted interventions to improve nurse–patient interactions and enhance care quality. **Conclusions:** The study highlights the impact of various factors on effective communication between nurses and patients, emphasizing the need for nurses to develop their communication skills and to receive adequate training from nursing officials.

## 1. Introduction

Effective communication between nurses and patients is crucial for providing high-quality healthcare, particularly when patients originate from various cultural backgrounds. Recognition of and respect for cultural differences play a significant role in treatment planning, fostering a positive nurse–patient relationship, and reducing communication obstacles [1]. Effective communication not only boosts patient satisfaction but also enhances physiological outcomes, adherence to treatment, and collaboration among healthcare professionals [2].

Nevertheless, miscommunication between nurses and patients from diverse cultural backgrounds can result in misunderstandings, incorrect diagnoses, and suboptimal care [3]. Elements such as language barriers, cultural beliefs, gender differences, religious practices, and social norms frequently present considerable challenges in nurse–patient interactions [4]. It is essential to address these challenges to enhance care delivery and foster cultural competence, which refers to the ability to operate effectively within the patient’s cultural framework [5].

Furthermore, language remains a significant barrier in nursing communication, particularly in multicultural environments. A recent investigation by Lee et al. (2023) revealed that language discrepancies were directly associated with delays in care and patient dissatisfaction [6]. Furthermore, the complexity of cultural competence necessitates ongoing training for healthcare professionals to equip them with the necessary skills to effectively navigate these barriers [7]. In Saudi Arabia, research conducted by Falatah et al. (2022) and Alhamad and Al-Qunaibet (2021) highlighted that nurses frequently encounter challenges in communicating with patients due to cultural and linguistic obstacles; this is a situation that appears to be exacerbated by the increasing number of expatriate workers in healthcare settings [8,9].

In addition to language, other factors, such as heavy workloads, elevated patient expectations, and a lack of familiarity with local customs, further hinder effective communication [10]. Recent studies have suggested various solutions, including the incorporation of cultural competency training within nursing education and the implementation of technological resources, such as interpreters and translation applications, to address communication barriers [11]. Ineffective communication, if not addressed, can lead to significant medical errors, with 80% of such errors attributed to communication failures [12].

Numerous studies indicate that language barriers are among the most significant obstacles to effective nurse–patient communication, especially in Saudi Arabia, where a substantial proportion of nurses are expatriates [8]. Saudi Arabia is characterized by its multiculturalism and acknowledged cultural diversity. Within the religious framework of the country, nurses are prohibited from making eye contact with or physically touching patients of the opposite sex, except in emergency situations [13]. Furthermore, while Arabic is the official language of Saudi Arabia, the majority of employed nurses are expatriates who communicate in English, which may create communication challenges between nurses and patients. Barriers to communication, such as language, religious and cultural norms, role modeling, and patients’ prior experiences, could significantly affect the nurse–patient communication and in turn affect the quality of patient care [14].

However, the cultural factors influencing nurse–patient communication in Saudi Arabia share similarities with those in other nations. Notably, religion plays a pivotal role in shaping healthcare practices and decisions related to patient care. Islamic perspectives on health, illness, and the human body significantly affect the types of treatments that patients may accept, thereby influencing their preferences for care and family involvement in decision making [15]. Family participation is crucial, particularly for female or elderly patients, as family members often take a leading role in healthcare choices. Nurses must adeptly navigate these familial dynamics to facilitate effective communication and provide appropriate care [16]. Language and dialect variations present considerable challenges within Saudi healthcare environments. Although Arabic is the official language, the existence of regional dialects and language barriers between expatriate nurses and patients can result in misunderstandings and dissatisfaction among patients [17]. Furthermore, gender norms can impact communication, as patients may prefer to receive care from nurses of the same gender, which can influence their comfort levels and the dynamics of communication [18]. Moreover, the coexistence of traditional medicine with Western healthcare practices can complicate communication. Patients may pursue alternative therapies, which could lead to non-adherence to the prescribed care plans, necessitating cultural sensitivity from nurses to effectively bridge this divide [19]. By addressing these cultural elements, including religion, family dynamics, language, gender norms, and alternative medical practices, nurse–patient communication in Saudi Arabia can be significantly improved. It is essential to integrate these cultural considerations into nursing education and training to enhance care quality and patient satisfaction in diverse healthcare contexts.

This study aimed to address a gap in the literature by identifying the factors and barriers affecting nurse–patient communication in a multicultural context, particularly in Jeddah, Saudi Arabia. Despite the growing recognition of the importance of effective communication in healthcare, there has been limited research on the barriers and factors affecting nurses’ communication when providing care for patients from diverse cultural backgrounds in the context of Saudi Arabia. This study aims to assess the factors and barriers affecting nurses’ communication when providing care for patients from diverse cultural backgrounds in Jeddah, Saudi Arabia. The following research questions are addressed:

1. What are the factors affecting nurses’ communication when providing care for patients from diverse cultural backgrounds in Jeddah, Saudi Arabia?
2. What are the barriers affecting nurses’ communication when providing care for patients from diverse cultural backgrounds in Jeddah?

In this study, the cultural competence model developed by Campinha-Bacote (2002) served as the theoretical foundation for the communication barriers and factors within a culturally diverse healthcare environment. This model emphasizes the need for healthcare providers to cultivate cultural awareness, knowledge, skills, and attitudes to improve their effectiveness in communicating with patients from various cultural backgrounds [20].

## 2. Materials and Methods

### 2.1. Study Design

A cross-sectional quantitative descriptive design using an online survey instrument was used in this study. The study employed this design to assess the factors and barriers affecting nurses’ communication when providing care for patients with a variety of cultural backgrounds in Jeddah. Descriptive studies are used to characterize people, events, or situations by examining natural themes [21]. Cross-sectional data collection methods provide an opportunity to compare and identify natural groups, like those based on gender, age, or education [22].

### 2.2. Study Setting and Participants

This research study is associated with the Faculty of Nursing at King Abdulaziz University in Jeddah, Saudi Arabia. Data were gathered through an online survey and distributed to registered nurses working in Jeddah’s hospitals in the city of Jeddah through social media platforms like WhatsApp and Twitter.

The target population for this study was registered nurses working in Jeddah’s hospitals. The inclusion criteria were as follows: registered nurses working in surgical or medical departments, outpatient clinics, critical care departments, and operating rooms in Jeddah’s hospitals; any nationality; either gender; an age of 20 years or above, with one year of experience or more. A convenience sample was used for the data collection. Convenience sampling is frequently employed in research settings where time limitations and accessibility issues hinder the application of more intricate sampling techniques; in these settings, it is a practical and economical choice [23]. This method is especially beneficial in exploratory research and in contexts involving populations that are difficult to access [24]. Although the results obtained may lack generalizability, they offer significant preliminary insights that can guide subsequent, more thorough investigations.

The total population comprises 7337 nurses in the Ministry of Health in Jeddah, and the calculated sample size was 366 nurses; according to the calculation by Raosoft, (version 4.5) the margin of error was confirmed to be 5%, and the confidence level was 95%. Participation was voluntary, and anonymity and confidentiality were assured.

### 2.3. Data Collection Method

The questionnaire was adopted from a previous study conducted by Norouzinia et al. (2015) [25].

The first part of the questionnaire was developed according to the purpose of study. It consisted of 8 items about sociodemographic factors: age, gender, nationality, educational level, total years of professional nursing experience, employment status, level of interest in the nursing profession, and courses related to communication skills.

The second part included 33 multiple-choice questions about the factors and barriers that limit communication between the nurse and the patient. The questions assessed the following:

- Factors associated with the nurse: lack of awareness of communication skills, negative attitudes, lack of self-confidence, frustration with the profession, and unwillingness to communicate.
- Patient-related factors: lack of awareness of nurses’ duties, negative attitudes, resistance to communication, lack of interest or concentration, anxiety, and physical discomfort.
- Organizational and environmental factors: lack of support from nursing administrators, lack of communication training, heavy workloads, inappropriate work environments, and the presence of critically ill patients.
- Personal factors: unpleasant experiences from previous encounters, lack of cooperation of patient companions, and interference by companions; these were adopted from Norouzinia et al. (2015) [25]. In addition, answers are given using a three-point Likert scale. A three-point Likert scale provides respondents with two options at the extremes and a neutral option in the middle; Agree, Disagree, and Neutral are examples of these options. This type of scale is less discriminating; so, it is used only when a wide range of emotional responses is not expected.

Data were collected through an online survey. An electronic link was used to gather the data. The data were gathered via an electronic link. Following ethical approval, the link was distributed via social media such as WhatsApp and Twitter. The participants were invited to participate in this study as well. Prior to completing the questionnaire, informed consent was obtained electronically. Participation was entirely voluntary and confidential. The researchers safeguarded the anonymous data and used it only for the purposes of this study. It took between 5 and 7 min to complete the questionnaires.

The study tool’s reliability and validity were assessed and validated. The adoption of the questionnaire from a previous study (Norouzinia et al., 2015) [25] ensured the validity of the instrument, as it had been previously used and tested. In addition, the content validity was established through the evaluation of three academic experts in the field of nursing at the faculty of nursing in King Abdulaziz University, Jeddah, to ensure the appropriateness of the questions. In addition, Cronbach’s alpha test was used to test the reliability of the tool.

### 2.4. Data Analysis

The most recent version of the Statistical Package for the Social Sciences (SPSS version 21) was used for data entry and analysis. Descriptive statistics were used in this study, including frequencies, mean, standard deviation, range, and percentages, to analyze the descriptive variables. In addition, the reliability of the questionnaire was tested using Cronbach’s alpha test.

### 2.5. Ethical Consideration

Ethical approval was granted by the ethical committee of the Faculty of Nursing at King Abdulaziz University, Saudi Arabia, on 13 December 2023 (NREC Serial No.: Ref No. 2B. 54). Furthermore, this study did not cause harm to the participants, and their rights were respected and protected. To protect the participants’ confidentiality, all the collected information was kept anonymous and kept safe in a locked cupboard in the researcher’s office, and the computer was locked with a password. Informed consent was obtained from all the participants before data collection. The participants were informed that participation was completely voluntary and that they had the right to withdraw at any time they wanted.

## 3. Results

### 3.1. Sociodemographic Characteristics

The findings presented in Table 1 are sociodemographic. With a sample size of 366 participants, the majority fell under the 20–24 age bracket, with an impressive representation of 38%, reflecting the youthful workforce in the nursing profession.

The study shows that 45% of the participants held a diploma, while 37% had a bachelor’s degree. The majority, 38%, had professional nursing experience ranging from 1 to 5 years, indicating that a considerable number were in the early stages of their nursing careers. The most common area of expertise was critical care, with a significant representation of 43% of the sample, where effective communication is particularly crucial due to the high-stakes nature of patient care. Female nurses dominated the sample size at 76%, reflecting the gender distribution commonly observed in the nursing profession. Additionally, Saudi nationals made up the majority at 54%, while 46% were non-Saudis, highlighting the multicultural composition of the nursing workforce in Jeddah. It is worth noting that the level of interest in the nursing profession was high, with 51% of participants expressing their passion for the nursing field. Furthermore, a substantial proportion, 73%, had attended courses on communication skills, indicating an awareness of the importance of effective communication in nursing practice.

### 3.2. Identifying Factors and Barriers in Effective Communication Between Nurses and Patients

To identify the factors and barriers related to the effective communication between nurses and patients and the nurses’ use of communication skills in interacting with patients, we calculated the statistical measures (mean and standard deviation), as shown in Table 2.

As shown in Table 2, the mean score of the factors and barriers was (2.84), which was above average; the S.dev was (0.456), indicating a significant presence of these challenges. The mean score was between (2.77 and 2.9), and the standard deviation was between (0.362 and 551).

The study found that the mean score and standard deviation of the age difference between the nurses and patients were 2.77 and 0.551, indicating a slight barrier that could have an impact on nurse–patient communication. Similarly, the mean score and standard deviation of the gender difference between the nurses and patients were 2.84 and 0.437; thus, this emerged as a significant barrier. The study also revealed that the mean score and standard deviation of the cultural difference between the nurse and the patient were 2.83 and 0.503, and the mean score and standard deviation of the religious difference between the nurse and the patient were 2.81 and 0.504; these findings indicated a moderate barrier and highlighted the potential challenges that can arise when nurses and patients have different cultural and religious backgrounds, as their beliefs, values, and expectations may differ, impacting their ability to communicate effectively. The study also showed that language differences between nurses and patients emerged as a prominent and highly significant barrier with a mean score of 2.87 and a standard deviation of 0.414. This finding suggests that differences in the languages spoken by nurses and patients can pose a significant obstacle to effective communication, potentially leading to misunderstandings and compromising the quality of care.

According to the results, the mean score and standard deviation of frustration and indifference among nurses towards their profession were 2.84 and 0.467; these were found to be significant barriers. The mean score and standard deviation of the nurses’ lack of awareness of the concept of communication and its types and of communication skills were 2.85 and 0.451; these were found to impact effective communication significantly. Furthermore, the mean score and standard deviation of the nurses’ lack of awareness of their verbal and non-verbal behaviors were 2.84 and 0.465, respectively. The mean score and standard deviation of the self-confidence among the nurses were 2.82 and 0.505, respectively. The mean score and standard deviation of the nurses’ negative attitude towards the patients were 2.82 and 0.49, respectively. The mean score and standard deviation of the nurses’ unwillingness to communicate with the patients were 2.8 and 0.531, respectively. These results suggest that the nurses’ own perceptions, attitudes, and confidence levels can significantly influence their ability to communicate effectively with patients.

The mean score and standard deviation of the nurses being very busy during the day in the ward were 2.87 and 0.398, and the mean score and standard deviation of the nurses not having enough time and opportunity were 2.87 and 0.397; these findings indicate that busy nurses and workload have an impact on effective communication with patients and contribute to poor nurse–patient interaction.

Finally, according to the results, the mean score and standard deviation of the family problems of the nurses were 2.8 and 0.507; these were found to affect the nurses’ ability to communicate effectively with patients. Additionally, the mean score and standard deviation for not receiving support from nursing managers in the field of maintaining communication skills were 2.84 and 0.465, respectively.

Moreover, the mean score and standard deviation of lack of sufficient training in communication principles and skills for nurses during the education period were 2.86 and 0.428 respectively, indicating that lack of nursing management support and training hinder nurse–patient communication. The mean score and standard deviation of the patient’s lack of awareness of the situation and the description of the nurse’s duties were 2.88 and 0.404 respectively. These findings indicate that all these factors can also create obstacles in effective nurse–patient communication.

The mean score and standard deviation of the patient’s negative attitude towards the nurse were 2.89 and 0.378; these findings were identified as a significant barrier to effective communication. Finally, the mean score and standard deviation of the resistance and reluctance of the patients to communicate were 2.84 and 0.431, respectively, and posed significant barriers. 

In addition, the mean score and standard deviation of the patient’s physical condition and the inability to speak or hear were 2.87 and 0.419; this indicates that patients who are physically unwell may have difficulty communicating clearly, which causes a barrier to communication. Furthermore, the mean score and standard deviation of the lack of necessary cooperation of the patient’s companions were 2.85 and 0.426; these findings are considered to be a significant barrier. Additionally, the mean score and standard deviation of the presence of the patient in the unfamiliar environment of the hospital were 2.83 and 0.508; this was found to cause a barrier and to hinder nurse–patient communication. In addition, the mean score and standard deviation of the busy environment (lots of noise and traffic) were 2.87 and 0.436; this reflects the fact that a busy and noisy environment is considered to be another major barrier.

Based on the results, the study identified a wide range of internal and external factors that were considered to be barriers to effective communication between nurses and patients, including demographic, linguistic, and cultural factors, as well as psychological factors. A key finding is that language differences are considered to be the prominent significant barrier, alongside cultural and religious differences. Additionally, nurse-related factors, such as attitudes, confidence, and workload, contributed significantly to the communication obstacles. Furthermore, lack of managerial support and insufficient training in communication skills among nurses were also identified as barriers. Finally, patient-related factors, such as negative attitudes, physical conditions, and the hospital environment, also had an impact on the hindering of communication. Additionally, it was found that the following factors were also barriers to effective communication between nurses and patients: lack of nurses in relation to patients; the patient’s lack of awareness of the situation and the description of the nurse’s duties; the nurse’s insufficient understanding of the patient’s needs and condition; the communication of other members of the health team with the nurse; the nurse was very busy during the day in the ward; the nurse did not have enough time and opportunity; the patient’s inability to speak or hear; the misinterpretation by the patient caused by his personal beliefs and values; the presence of a critically ill patient in the ward (because most of the nurse’s time is spent providing services to this patient; and, finally, the lack of nurse awareness of the concept of communication and its types and of communication skills.

The findings of the study showed that various factors can affect effective communication between nurses and patients. Therefore, nurses should develop their communication skills to establish a positive relationship with patients. Nursing officials should provide adequate training and support to address these factors and enhance nurses’ communication skills for better patient care.

## 4. Discussion

Effective communication between nurses and patients plays a crucial role in the delivery of quality healthcare services. It fosters trust, understanding, and collaboration and ultimately contributes to better health outcomes and patient satisfaction. This quantitative study aimed to assess the factors and barriers affecting nurses’ communication when providing care for patients from diverse cultural backgrounds in Jeddah, Saudi Arabia, a city renowned for its rich cultural diversity.

The study involved a sample of 367 participants, which predominantly comprised young nurses aged between 20 and 24 years (38%), reflecting the youthful workforce in the nursing profession. A significant portion of the participants (45%) held a diploma in nursing, while 37% had a bachelor’s degree. The majority (38%) had between 1 and 5 years of professional experience, indicating that a considerable number were in the early stages of their nursing careers.

Notably, the largest group of participants (43%) worked in critical care settings, where effective communication is particularly crucial due to the high-stakes nature of the patient care. The study sample was predominantly female (76%), reflecting the gender distribution commonly observed in the nursing profession. Additionally, Saudi nationals constituted 54% of the participants, while 46% were non-Saudis, highlighting the multicultural composition of the nursing workforce in Jeddah.

It is worth noting that a significant proportion (51%) of the participants expressed a high level of interest in the nursing profession, reflecting their passion and commitment to their chosen career path. Furthermore, a substantial proportion (73%) had attended courses on communication skills, indicating an awareness of the importance of effective communication in nursing practice.

The primary objective of this quantitative study was to identify and assess the factors and barriers that influence effective communication between nurses and patients from different cultural backgrounds in Jeddah. The study employed a questionnaire as the primary data collection instrument to assess the factors and barriers affecting nurse–patient communication. The questionnaire consisted of two parts.

The first part gathered demographic information about the participants, including age, gender, nationality, educational level, years of professional nursing experience, department of work, employment status, level of interest in the nursing profession, and whether they had attended communication skills courses.

The second part of the questionnaire focused on identifying the factors of and barriers to effective communication between nurses and patients. This section included multiple-choice questions with responses given using a three-point Likert scale (Agree, Disagree, and Neutral). The questions were adopted from a previous study by Norouzinia et al. (2015), which explored the communication barriers perceived by nurses and patients [25]. The questionnaire covered a comprehensive range of potential factors and barriers, including demographic factors, nurse-related factors, patient-related factors, organizational and environmental factors, and interpersonal factors.

The study revealed several key findings. The mean score of the factors and barriers affecting nurse–patient communication was 2.84 (on a three-point scale), indicating a significant presence of these challenges. In addition, language differences between the nurses and patients emerged as a prominent barrier, with a mean score of 2.87. This finding suggests that differences in language between the nurses and patients posed a significant obstacle to effective communication. This situation resulted in misunderstandings, a lack of trust, and compromised patient care. When there is no shared language, critical information may be lost, misinterpreted, or inadequately communicated, which can lead to adverse outcomes, such as patient dissatisfaction or inappropriate treatment. Language proficiency is a vital demographic factor impacting communication; nurses who lack proficiency in the local dialect or the patients’ language may experience significant challenges in delivering effective services.

In addition, the study found that the mean score of the age difference between the nurses and patients was 2.77, indicating a slight barrier to nurse–patient communication, and the mean score of the gender difference between the nurses and patients was 2.84, indicating a significant barrier to the communication between the nurses and patients. Thus, the individual demographics of patients also influence communication, as factors such as age, education level, and language proficiency can affect interactions. For example, patients with lower educational backgrounds may struggle to comprehend certain medical terminology or treatment options, leading to misunderstandings and dissatisfaction. Furthermore, the gender and cultural orientation of patients can shape how they receive and interpret information. Cultural norms and family dynamics often play a crucial role in how patients express their healthcare needs; thus, cultural competence is essential for providing effective care. Comprehending these dynamics significantly contributes to the enhancement of communication between nurses and patients, ultimately leading to improved healthcare outcomes.

Moreover, cultural and religious differences were also identified as significant factors, with mean scores of 2.83 and 2.81, respectively. These findings highlight the potential challenges that can arise when nurses and patients have different cultural and religious backgrounds, as their beliefs, values, and expectations may differ, impacting their ability to communicate effectively; the findings emphasize the influence of diverse cultural norms, beliefs, and practices. These variations created difficulties in establishing rapport, fostering patient trust, and aligning expectations regarding healthcare delivery. For instance, specific cultural practices or religious beliefs may affect how patients react to medical treatments, thereby influencing their willingness to engage in open communication with nurses. This can result in a lack of collaboration and diminished patient satisfaction.

However, factors related to nurses, such as lack of awareness about communication skills (mean score 2.85), negative attitudes (mean score 2.82), and lack of self-confidence (mean score 2.82), were found to impact effective communication. These results suggest that the nurses’ own perceptions, attitudes, and confidence levels can significantly influence their ability to communicate effectively with patients. Patient-related factors, including lack of awareness of nurses’ duties (mean score 2.88), negative attitudes (mean score 2.89), and resistance to communication (mean score 2.84), also posed barriers. These findings indicate that patients’ perceptions, attitudes, and willingness to communicate can also create obstacles in effective nurse–patient communication. Negative attitudes can come from personal beliefs, dissatisfaction, or fatigue, which in turn hinder effective communication.

The findings of this study are consistent with several previous studies mentioned in the literature review and proposal. Norouzinia et al. (2015) identified language differences as a significant communication barrier between nurses and patients, which aligns with the current study’s findings [25]. Similarly, Albagawi (2014) used a mixed-methods approach to identify barriers and facilitators of nurses’ communication towards Arabic patients in Saudi Arabia and found that language barriers between expatriate nurses and patients who could not speak English represented a significant barrier affecting nurse–patient communication [13]. In contrast, Barilaro et al. (2019) reported no significant relationship between gender, education level, and satisfaction with nursing communication; this was in contrast to the current study’s findings, which identified gender and cultural differences as factors affecting communication [4]. The discrepancies could be attributed to variations in study settings and cultural contexts.

Falatah et al. (2022) found a significant correlation between cultural competency and structural empowerment among nurses in Saudi Arabia, supporting the current study’s findings that cultural differences can impact nurse–patient communication [8]. Paredath et al. (2023) highlighted language barriers as a significant challenge for nurses during the Hajj season in Saudi Arabia, which was consistent with the current study’s results [6].

This study provides valuable insights into the factors and barriers affecting nurse–patient communication in a multicultural setting like Jeddah, Saudi Arabia. The findings highlight the importance of addressing language barriers, promoting cultural awareness and competency, and enhancing nurses’ communication skills.

Regarding language differences, the high mean score of 2.87 indicated that language differences pose a significant barrier to effective communication between nurses and patients. This finding aligns with the study by Bit-Lian et al. (2020), which recognized that differences in language and accent can create obstacles in nurse–patient communication [5].

When nurses and patients do not share a common language or have difficulties understanding each other’s language or accent, it can lead to misunderstandings, miscommunication, and challenges in conveying important information accurately. This can impact the quality of care, patient satisfaction, and the ability to address the patient’s concerns effectively.

Similarly, cultural and religious differences were identified as significant factors, with mean scores of 2.83 and 2.81, respectively, on a three-point scale, indicating a considerably high level of impact. The study emphasizes the need for cultural competency training and strategies to bridge cultural gaps, as nurses and patients may have different perspectives and expectations shaped by their backgrounds. For example, a nurse’s cultural beliefs and values may differ from those of a patient, leading to misunderstandings or difficulties in effectively conveying information or addressing the patient’s concerns. These findings align with Bit-Lian et al. (2020), who reported that cultural and religious differences along with customs can be obstacles to effective nurse–patient communication [5].

In addition to language and cultural barriers, age differences between nurses and patients emerged as a barrier, with a mean score of 2.77, indicating a substantial impact. This finding is supported by Norouzinia et al. (2015), who found that the age difference between nurses and patients was perceived as a barrier to the use of communication skills by nurses in interacting with patients [25].

Gender differences between nurses and patients also emerged as a significant barrier, with a mean score of 2.84 on a three-point scale, suggesting a notable impact. This finding supports the assertion by Bit-Lian et al. (2020) that gender differences can pose challenges in nurse–patient communication [5]. The high mean score of 2.84 suggests that gender dynamics, such as potential gender biases, communication style differences, or cultural expectations related to gender roles, can create barriers to effective communication between nurses and patients.

Turning to nurse-related factors, lack of awareness about communication skills (mean score 2.85), negative attitudes (mean score 2.82), and lack of self-confidence (mean score 2.82) were also found to significantly impact effective communication. Negative attitudes towards patients, lack of awareness about communication skills, and low self-confidence among nurses can create barriers in understanding and responding to patients’ needs effectively. For instance, a nurse with a negative attitude towards a patient may fail to actively listen or respond empathetically, leading to poor communication and potential dissatisfaction with care. Barilaro et al. (2019) reported no significant relationship between nurses’ attitudes and satisfaction with nursing communication, which contrasts with the current study’s findings [4].

Furthermore, patient-related factors, including lack of awareness of nurses’ duties (mean score 2.88), negative attitudes (mean score 2.89), and resistance to communication (mean score 2.84), posed significant barriers. These elevated mean scores suggest a substantial impact. When patients are unaware of the roles and responsibilities of nurses, they may have unrealistic expectations or fail to collaborate effectively in their care. Additionally, negative attitudes or resistance to communication from patients can impede the exchange of information and hinder the establishment of a trusting and productive nurse–patient relationship. Barnawi and Barnawi (2023) found that patients’ ignorance of nurses’ status and responsibilities, as well as their physical pain, discomfort, and anxiety, were significant barriers to effective nurse–patient communication, aligning with the current study’s findings [2].

Finally, organizational and environmental factors, such as lack of support from nursing officials (mean score 2.84), absence of communication training (mean score 2.86), and unsuitable work environments (mean score 2.81), emerged as significant barriers. The elevated mean scores indicate a considerable impact. The study identified organizational and environmental challenges, such as inadequate communication training, excessive workloads, and inappropriate work settings, as major obstacles. These factors contributed to nurses feeling overwhelmed, preventing them from allocating adequate time for patient communication; in addition, they lacked the necessary support to overcome communication challenges. This situation led to heightened stress levels among nurses and diminished job satisfaction, adversely affecting the quality of interactions between nurses and patients. The findings emphasize the significance of cultural competence training in addressing the barriers identified in the study. By enhancing nurses’ cultural sensitivity and communication abilities, such training would enable nurses to more effectively manage cultural and linguistic differences. The anticipated outcome would be a marked improvement in nurse–patient communication, increased patient satisfaction, and, ultimately, better health outcomes for individuals from varied cultural backgrounds. Paredath et al. (2023) emphasized the need for transcultural nursing education and training to overcome cultural barriers, which is consistent with the current study’s findings regarding the importance of communication training [6].

The study highlights the need for cultural competency training, language support services, and ongoing professional development for nurses to improve their communication skills. It highlights the importance of addressing organizational and environmental factors that can impact nurse–patient interactions, ensuring supportive work environments and policies that facilitate effective communication.

Effective communication between nurses and patients is crucial for several reasons. First, it enables nurses to gather accurate and comprehensive information about a patient’s condition, concerns, and preferences, allowing more informed and personalized care planning. Second, it fosters trust and rapport between the nurse and patient, which can enhance treatment adherence and overall patient satisfaction. Third, clear and empathetic communication can help alleviate patient anxiety, reduce misunderstandings, and promote better health outcomes.

Moreover, effective nurse–patient communication plays a vital role in patient safety and quality of care. Miscommunication or misunderstandings can lead to medication errors, missed follow-up appointments, or failure to adhere to prescribed treatments, potentially compromising patient well-being. By addressing the factors and barriers identified in this study, healthcare organizations can take proactive steps to improve communication, mitigate risks, and ensure the delivery of safe and high-quality care.

This study encountered limitations related to the data collection process, particularly in terms of obtaining a sufficient number of responses within the given time constraints through convenience sampling. However, the small sample size in this study may impact the statistical power and the ability to generalize the findings to a broader population. Therefore, to maintain the integrity of the findings of the study, this recruitment challenge, which is common in research, was carefully taken into consideration during the analysis stage in order to preserve the integrity of the study’s findings by obtaining the study’s power with the final sample size, thus guaranteeing that the findings were still reliable and significant. Furthermore, the study was carried out in a single setting in the geographical area of Jeddah in Saudi Arabia, which hindered the generalizability of the results beyond the specific context of the study. It is crucial for the subsequent research to be conducted across multiple sites and diverse settings. Therefore, future studies should utilize a random or stratified sampling method and expand the sample size to include patients and nurses from other locations and should use a mixed-methods approach for comprehensive data and the generalization of results.

Another limitation is the use of an online survey for data collection; it is important to note that self-reported data may be subject to biases, such as social desirability bias or recall bias, where the participants may provide responses that they deem socially acceptable, or they may have difficulty accurately recalling their experiences and their true feelings, particularly concerning sensitive topics related to cultural differences and communication barriers. Future studies could mitigate these biases by conducting different data collection methods, such as interviews.

Another significant limitation of this study is the use of a three-point Likert scale in the survey which may lack the sensitivity required to capture the complexity of responses, leading to an oversimplification of intricate participant attitudes and opinions, as well as limiting the richness of responses. Future research could address these limitations by diversifying the data collection methods and employing a more detailed response scale. A more effective approach might involve utilizing a five- or seven-point Likert scale, which would provide greater depth and nuance to the responses, thereby offering a clearer understanding of participants’ perspectives.

## 5. Conclusions

This study aimed to assess the factors and barriers affecting nurses’ communication when providing care for patients from diverse cultural backgrounds in Jeddah, Saudi Arabia. A cross-sectional quantitative descriptive design was employed using an online survey instrument. A convenience sample of 366 registered nurses employed in Jeddah’s hospitals were involved in the study. The study concluded that effective and interactive communication is necessary to achieve a satisfactory nurse–patient relationship. Research shows that it is the nurse’s job to encourage the development of this relationship by removing barriers that impede communication. The respondents considered communication impairments to be related to personal and social characteristics, work norms, the patient’s clinical situation, and environmental factors.

Important findings were made regarding cultural knowledge of the work environment in terms of the nurses’ perceptions, cultural norms, and life activity culture. Establishing a satisfactory nurse–patient relationship requires efficient and interactive communication. According to the research, it is the nurse’s job to make that happen and to build relationships by resolving the barriers that prevent communication. The main barrier is communication skills, which cannot be ignored as this is a core belief of the patient. There are also patient-related barriers that nurses cannot change, such as religious issues or education levels, but nurses must find ways to break down these barriers.

This study contributes to the existing body of knowledge on nurse–patient communication by assessing the factors and barriers affecting nursing communication in a multicultural environment, specifically in Jeddah, Saudi Arabia. It highlights several key challenges, including language barriers, cultural and religious influences, and nurse-related issues such as attitudes and self-confidence. The research expands upon the cultural competence model by exploring a broader array of factors, including organizational and interpersonal dynamics that impact communication in varied healthcare contexts. The focus on Jeddah’s diverse population, which has been relatively overlooked in communication studies, is particularly significant. This study emphasizes the necessity of cultural competency training to address language and cultural barriers, as well as organizational challenges like heavy workloads and insufficient support. These insights provide valuable strategies for enhancing communication in multicultural healthcare settings.

### Implications

The findings of this study have significant implications for healthcare providers, policymakers, and educators in Saudi Arabia and other multicultural settings.

Nursing Practice

There is a need for educational programs and training for both international and local nurses as they begin their employment in a multicultural environment to equip them with effective communication skills and increase their understanding of communication processes, thereby enabling them to provide high-quality care. In addition, it is recommended that an ongoing evaluation of course content is necessary to assess nurses’ comprehension and the appropriateness of cross-cultural communication. Additionally, resources that promote self-directed learning should be developed and made easily accessible to staff to meet the specific needs of their respective regions.

Nursing administration

The findings identified several communication barriers, such as excessive workloads, fatigue, lack of awareness, and uncommunicative attitudes of nurses. Therefore, it is essential for the health settings to provide sufficient support by managing nurses’ workloads effectively and ensuring job satisfaction. Creating a supportive environment, addressing patients’ emotional challenges, raising awareness among patients, and enhancing nurses’ communication skills are vital steps. Improved engagement by nurses can be fostered through the provision of resources and amenities that alleviate nurse fatigue, a reduction in working hours, and incentives for nurses who demonstrate exemplary communication skills. Additionally, the outcomes of this study can support nursing managers in making assessments of nursing personnel regarding communication competencies. It is also necessary to implement effective time management strategies for nurses, standardize the nurse-to-patient ratio, ensure a safe and comfortable environment, and limit the number of visitors or relatives present at any given time. Regarding policy, there should be a development and implementation of a standardized set of behavioral policies and procedures.

Nursing education

Nursing education must encompass content related to transcultural concepts, with a particular focus on communication within multicultural contexts. It is essential for nursing students to learn about various aspects of transcultural communication to deliver effective and efficient transcultural nursing care.

Nursing Research

This study primarily concentrated on nurses’ perspectives only. Understanding the patients’ perspectives is also crucial, as they are the recipients of care. Therefore, further studies need to explore the perspectives of patients regarding the barriers and facilitators of communication within a multicultural setting. Moreover, it is recommended to create new self-administered communication surveys in both Arabic and English, incorporating key subscales related to gender, religion, culture, and language. This would enable the capture of Arabic patients’ perspectives, facilitating the assessment of nurse–patient communication and the exploration of existing communication challenges in hospitals in the Kingdom of Saudi Arabia.

Moreover, this study offers significant contributions to the cultural competence model [20], especially regarding its implementation in healthcare environments marked by considerable cultural diversity. The model posits that successful healthcare delivery requires that providers cultivate specific competencies related to cultural awareness, knowledge, skills, and interactions. The findings of this study, which examines the barriers to nurse–patient communication in a multicultural setting in Jeddah, Saudi Arabia, provide empirical validation for essential elements of this model and demonstrate its practical applicability in nursing practice. The findings emphasize the vital need to tackle cultural and communication obstacles at both the individual and organizational tiers to enhance nurse–patient interactions within a multicultural framework. The cause-and-effect relationships identified in the study offer a comprehensive framework for comprehending the impact of cultural and interpersonal dynamics on communication results, while also proposing strategies to mitigate these challenges.

## Figures and Tables

**Table 1:** Sociodemographic characteristics.

Variable	N = 367	Frequency	Percent
Age	20–24 years	140	38
	25–29 years	58	16
	30–34 years	63	17
	Above 35	106	29
Educational Level	Diploma	165	45
	Bachelor’s Degree	135	37
	Master’s Degree	15	4
	PhD	52	14
Total years of professional nursing experience	1–5 years	141	38
	6–10 years	64	17
	11–15 years	65	18
	More than 15 years	97	26
Department field	Outpatient clinics	77	21
	Critical care	158	43
	Internal medicine or surgery	74	20
	Operating room	58	16
Gender	Male	89	24
	Female	278	76
Nationality	Saudi	199	54
	Non-Saudi	168	46
Shift work	Rotating	204	56
	Fixed at night	16	4
	Fixed in the morning	125	34
	Fixed in the evening	22	6
Employment status	Employee	106	29
	Unemployed	74	20
	Retired	187	51
The level of interest in the nursing profession	Low	18	5
	Medium	165	45
	High	186	51
Have you attended courses on communication skills?	Yes	267	73
	No	100	27
Do you have a job or work other than nursing?	Yes	97	26
	No	270	74

**Table 2:** The mean score and standard deviation of factors and barriers in effective communication between nurses and patients.

Item	Mean	Standard Deviation
Age difference between nurse and patient:	2.77	0.551
Gender difference between nurse and patient:	2.84	0.437
The cultural difference between the nurse and the patient:	2.83	0.503
The religious difference between the nurse and the patient:	2.81	0.504
The difference in the language of conversation between the nurse and the patient:	2.87	0.414
Frustration and indifference among nurses towards their profession:	2.84	0.467
The nurse’s lack of awareness of the concept of communication, its types, and communication skills:	2.85	0.451
The nurse’s lack of awareness of her verbal and non-verbal behaviors:	2.84	0.465
Self-confidence among the nurses:	2.82	0.505
The nurse’s negative attitude towards the patient:	2.82	0.49
The nurse unwillingness to communicate with the patient:	2.8	0.531
The nurse’s insufficient understanding of the patient’s needs and condition:	2.87	0.417
Unpleasant experiences of nurses from previous encounters with patients:	2.85	0.469
The department where the nurse works:	2.8	0.518
Communication of other members of the health team with the nurse:	2.87	0.401
Lack of nurses in relation to patients:	2.9	0.362
The nurse is very busy during the day in the ward:	2.87	0.398
The nurse does not have enough time and opportunity:	2.87	0.397
Work planning is against the wishes of the nurse:	2.85	0.445
The nurse has several jobs, and fatigue is caused by extra work:	2.85	0.461
Problems and mental occupations of nurses outside of nursing work:	2.83	0.498
Physical problems and diseases of the nurse:	2.85	0.414
Family problems of the nurse:	2.8	0.507
Inadequate economic status of the nurse:	2.8	0.507
Lack of attention of nursing officials to the way nurses communicate with patients in periodical evaluations:	2.83	0.492
Not receiving support from nursing managers in the field of maintaining communication skills:	2.84	0.465
Absence of specific laws, principles, and standards for nurses in dealing with patients:	2.85	0.467
Lack of sufficient training in communication principles and skills given to nurses during the education period:	2.86	0.428
Failure to hold in-service training courses about communication and its skills for nurses:	2.86	0.424
The patient’s lack of awareness of the situation and description of the nurse’s duties:	2.88	0.404
The patient’s negative attitude towards the nurse:	2.89	0.378
Resistance and reluctance of the patient to communicate:	2.84	0.431
Lack of attention and concentration of the patient:	2.85	0.429
Anxiety and worry, pain, and physical discomfort of the patient:	2.84	0.456
The patient’s inability to speak or hear:	2.87	0.419
The misinterpretation by the patient caused by his personal beliefs and values:	2.87	0.384
Lack of necessary cooperation of the patient’s companions:	2.85	0.426
Many interventions of the patient’s companions:	2.83	0.452
The presence of the patient’s companions at the patient’s bedside:	2.8	0.513
The presence of the patient in the unfamiliar environment of the hospital:	2.83	0.508
Busy environment (lots of noise and traffic):	2.87	0.436
Unprincipled interactions of third-ranking nursing officials with nurses:	2.8	0.516
Unsuitable environmental conditions (lack of proper ventilation in the environment, heat and cold, inappropriate light, unpleasant odor, etc.):	2.81	0.501
The presence of a critically ill patient in the ward (because most of the nurses’ time is spent providing services to this patient):	2.86	0.432
Total Mean	2.84	0.456

## Data Availability

Data are available upon reasonable request from the corresponding author.
